# Gallomyrtucommulones G and H, New Phloroglucinol Glycosides, from Bioactive Fractions of *Myrtus communis* against *Staphylococcus* Species

**DOI:** 10.3390/molecules27207109

**Published:** 2022-10-21

**Authors:** Francesca Guzzo, Alexandra G. Durán, Cinzia Sanna, Rosangela Marasco, Nicola Molfetta, Elisabetta Buommino, Antonio Fiorentino, Brigida D’Abrosca

**Affiliations:** 1 Dipartimento di Scienze e Tecnologie Ambientali Biologiche e Farmaceutiche–DiSTABiF, Università degli Studi della Campania “Luigi Vanvitelli”, Via Vivaldi 43, 81100 Caserta, Italy; 2 Allelopathy Group, Department of Organic Chemistry, Institute of Biomolecules (INBIO), School of Science, University of Cadiz, Campus de Excelencia Internacional (ceiA3), C/ República Saharaui n° 7, 11510 Puerto Real, Spain; 3 Department of Life and Environmental Sciences, University of Cagliari, Via Sant’Ignazio da Laconi 13, 09123 Cagliari, Italy; 4 Dipartimento di Farmacia, Università degli Studi di Napoli “Federico II”, Via Domenico Montesano 49, 80131 Napoli, Italy

**Keywords:** *Myrtus communis*, gallomyrtucommulone, structural elucidation, NMR analysis, antimicrobial assay, *Staphylococcus epidermidis*, *Staphylococcus aureus* MRSA

## Abstract

Myrtaceae family is a continuous source of antimicrobial agents. In the search for novel antimicrobial agents against *Staphylococcus* species, bioactive fractions of *Myrtus communis* L., growing in the Sardinia island (Italy) have been investigated. Their phytochemical analysis led us to isolate and characterize four alkylphloroglucinol glycosides (**1**–**4**), three of them gallomyrtucommulones G–H (**1**,**2**), and myrtucommulonoside (**4**) isolated and characterized for the first time. The structures of the new and known compounds, endopreroxide G3 (**5**), myricetin-3-*O*-glycosides (**6**,**7**) were determined based on the spectroscopic evidence including 1D-/2D-NMR and HR-MS spectrometry. Enriched fractions as well as pure compounds were tested for their antimicrobial activity by broth micro-dilution assay against *Staphylococcus epidermidis* and *S. aureus.* Results reported herein demonstrated that gallomyrtucommulone G (**1**) showed a selective antimicrobial activity against both *S. aureus* strains (ATCC 29213 and 43300) until 16 μg/mL while gallomyrtucommulone D (**3**) showed the best growth inhibition value at 64 μg/mL.

## 1. Introduction

The skin is populated by different genera of bacteria and yeasts, coexisting in the same microenvironment thanks to interspecies interaction ensuring the health of the host [1]. Among the Gram-positive bacteria, the main constituent of the normal flora on the human skin and mucous membranes is *Staphylococcus epidermidis,* a coagulase-negative *Staphylococcus* that contributes to host defense by avoiding the colonization from other potentially harmful microorganisms. It also plays an important role in the modulation of inflammation by increasing the skin’s antimicrobial defense [2]. Nevertheless, once the host epithelial barrier is compromised, *S. epidermidis* is responsible for nosocomial infection due to its ability to form biofilm on indwelling medical devices, causing prosthetic joint, vascular graft, surgical site, central nervous system shunt and cardiac device infections [3,4]. Moreover, the skin microbiota can also be colonized by pathogenic strains, such as *S. aureus,* the main cause of both community- and hospital-acquired infections. It can colonize almost every tissue and organ, as well as implants, and can frequently manifest resistance to antibiotic therapy, a serious problem that is involving researchers worldwide. The emergence of multi-drug resistant strains such as MRSA (methicillin-resistant *S. aureus*) is nullifying the efforts made until now, virtually eliminating the use of β-lactams as therapeutic options against *S. aureus* [5].

Among natural sources, plants represent a valuable source of a wide spectrum of specialized metabolites, which have a vast variety of biological activities including antimicrobial properties [6,7]. In this context, the Myrtaceae family, the ninth largest family of flowering plants and known for their high biodiversity, is a source of antimicrobial agents against pathogenic microorganisms [8,9]. Callistrilone A from *Callistemon rigidus* [10], rhodomyrtone A from *Rhodormytus tomentosa* [11], eugenials C and D from *Eugenia umbelliflora* [12], and myrtocummulone A from *Myrtus communis* L. [13] are the most common phloroglucinols derivatives that have been reported for their good activity against different strains of *S. aureus* MRSA. In particular, *M. communis* L. (Myrtaceae), a Mediterranean evergreen shrub growing in the wild in hill regions from Morocco to Iran, is an important source of myrtocummulones derivatives [14,15], while alkylphloroglucinol glycosides are less prevalent [16]. This species has been used since ancient times as an ornamental plant and as a source of fragrance and medications. Furthermore, *M. communis* has displayed anti-inflammatory, antiseptic, antimicrobial, and hypoglycemic properties [15]. In pursuing our investigation of plants growing in the Sardinia region [17], endowed of peculiar chemical profiles [18], the bio-guided phytochemical investigation of glycosidic fractions of *M. communis* leaves was performed. Herein we describe the isolation, from active fractions against *Staphylococcus epidermidis*, and the structural characterization by 2D-NMR of two new galloylated alkylphloroglucinol glucosides (Gallomyrtucommulones G–H, **1**–**2**), one alkylphloroglucinol glucosides (Myrtucommulonoside, **4**) along four known compounds. The antimicrobial properties of the enriched fractions and pure compounds have been also assessed.

## 2. Results and Discussion

### 2.1. Antimicrobial Activity of Hydroalcholic Crude Extract and First Purification Step

The crude extract of *M. communis* leaves (MC) was tested for antimicrobial capacities on two strains of *S. epidermidis*: *S. epidermidis* ATCC 12228 (control strain) and *S. epidermidis* ATCC 35984 (biofilm producer strain). MC showed a discrete antimicrobial activity on both strains resulting more active on *S. epidermidis* ATCC 35984. In fact, at 32 µg/mL MC reduced by 45.6% ± 4.8 the growth of the strain biofilm producer (ATCC 35984), while at the same concentration the inhibition of the growth of the control strain (ATCC 12228) was 26.7% ± 2.1. However, a discrete antimicrobial activity (31.3% ± 2.8) against *S. epidermidis* ATCC 35984 was evident until 2 µg/mL. The positive control, vancomycin, strongly inhibited the growth of *S. epidermidis* at 2 µg/mL (data not shown). By considering the promising results, MC was further chromatographed on Amberlite XAD-4 and the methanolic fraction was separated by CC-flash chromatography furnishing three fractions (MC_A-MC_C) from less polar MC_A to more polar MC_C. The fractions were characterized by different metabolites contents as highlighted by their ^1^H-NMR profiling. In particular, MC_A showed prevailing signals in the aliphatic region (1–6–1.2 ppm) as six methyl singlets besides one singlet in the aromatic region. The ^1^H NMR spectrum of MC_B fraction showed resonances typical of gallic acid (δ_H_ 7.10) as well as a cluster of olefinic and oxymethine singlets in the range between 5.0 and 3.9 ppm, and a 6-acylated glycosyl moiety. The fine splitting of all signals in the upfield region of the spectrum suggested a mixture of other related compounds. Finally, MC_C was constituted almost exclusively by myricitrin [19]. 

**Figure 1:**
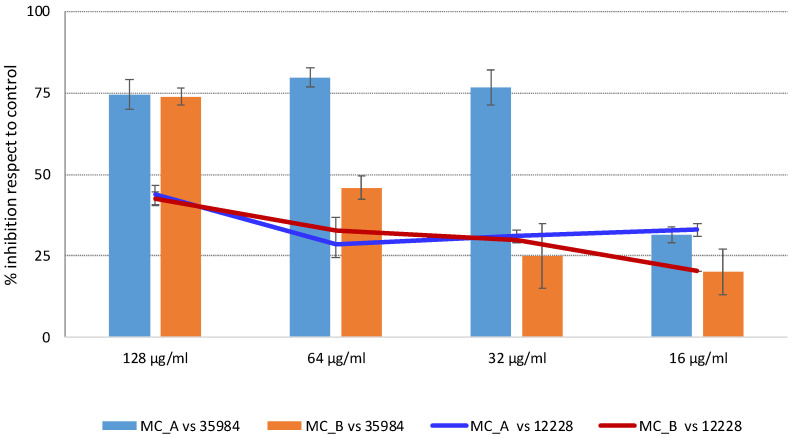
Antimicrobial assay of MC_A and MC_B fractions against *S. epidermidis* 12228 and 35984 strains.

All the three above mentioned fractions (MC_A-MC_C) were tested against both *S. epidermidis* strains (Figure 1). Myricitrin turned out to be inactive (data not shown), while MC_A showed the best activity on *S. epidermidis* ATCC 35984, reducing the growth by 77%, until the concentration of 32 µg/mL. At the same concentration, MC_B reduced by only 25% the growth of the same bacterial strain. The similar trend has been revealed against *S. epidermidis* ATCC 12228. Based on the promising results, MC_A and MC_B were then chosen for a more in-depth phytochemical study. 

### 2.2. Fractionation of MC_A: Antimicrobial Assessment 

**Figure 2:**
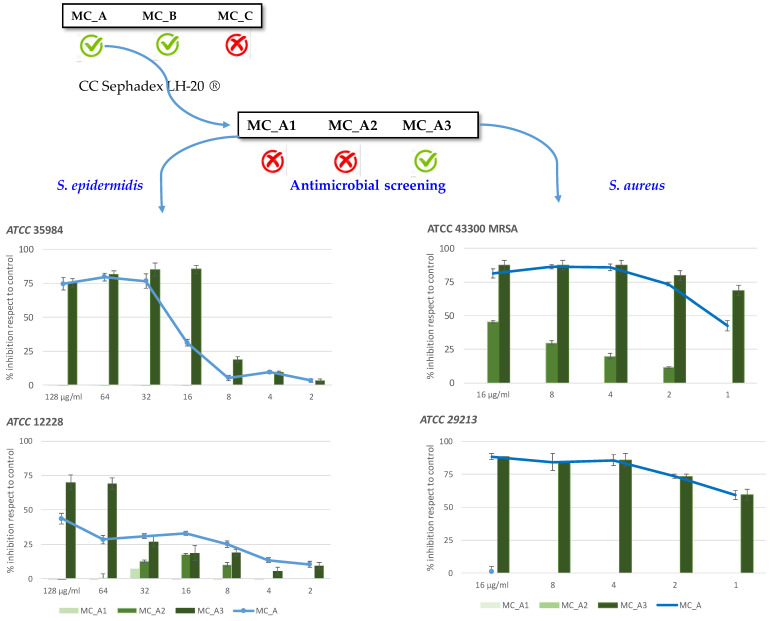
Purification and antimicrobial assessment of MC_A derived fractions (MC_A1, MC_A2, MC_A3) against *S. epidermidis* and *S. aureus* strains.

The interesting results obtained for the less polar fraction (MC_A) of crude extract prompted us to go on its purification. Three fractions, MC_A1, MC_A2 and MC_A3, were obtained and tested against both *S. epidermidis* strains until here used (Figure 2). 

Only fraction MC_A3 turned out to be active. In fact, MC_A3 reduced by 70% the growth of *S. epidermidis* ATCC 12228 at 128 μg/mL, and against *S. epidermidis* ATCC 35984 induced a stronger percentage of growth inhibition (84.5%) at 16 μg/mL (Figure 2). MC_A1 and MC_A2 did not inhibit the growth of *S. epidermidis* ATCC 35984.

Based on the interesting results obtained, all the fractions MC_A1, MC_A2, MC_A3, were assayed against the pathogenic strains of *S. aureus* ATCC 29213 and *S. aureus* ATCC 43300 (methicillin-resistant strain). The most active fraction turned out to be MC_A3, which showed the best activity against both *S. aureus* strains with a MIC value of 4 μg/mL (Figure 2). The positive control, oxacillin, was not able to inhibit the growth of *S. aureus* MRSA as efficiently as MC_A3 did (data not shown). On the contrary, MC_A1 did not inhibit the growth of both strains, while MC_A2 turned out to be scarcely active, reducing by 45% the growth of *S. aureus* ATCC 43300 at 16 μg/mL (Figure 2).

**Figure 3:**
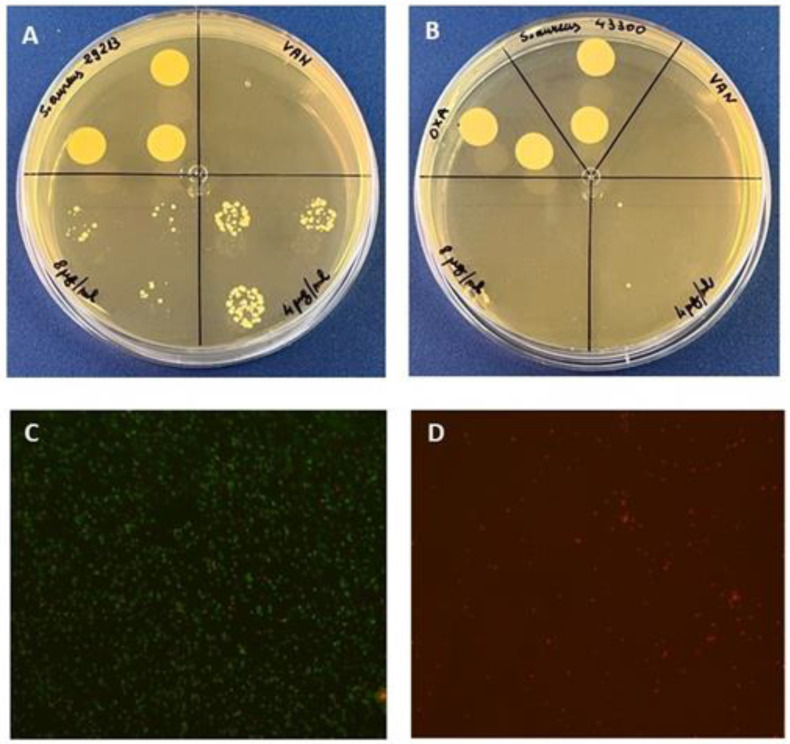
Bactericidal activity of MC_A3 (panels (**A**,**B**)). Representative pictures of live/dead imaging of *S. aureus* MRSA after 4 μg/mL MC_A3 treatment (panels (**C**,**D**)). Cells were observed at 20× magnification (Iris Digita System, Twin Helix). After 24 h, control cells showed uptake of only acridine orange (**C**) (green fluorescence), while MC_A3-treated cells showed uptake of ethidium bromide (**D**) (red fluorescence).

The activity of MC_A3 was also investigated in terms of bactericidal activity. The results, reported in Figure 3 (panel A), demonstrated the bactericidal activity at 8 and 4 μg/mL against both *S. aureus* strains, since it caused an over 3log_10_ -fold reduction in the bacterial count (2.9 × 10^3^ CFU/mL for 4 μg/mL treated-*S. aureus* ATCC 12228 versus 1.8 × 10^8^ CFU/mL for untreated control cells), whereas no colonies’ growth was observed against the MRSA at both concentrations (panel B). Live/dead imaging of *S. aureus* MRSA treated at the MIC values confirmed the latest results (Figure 3, panels C,D). MC_A3-treated-*S. aureus* showed an uptake of ethidium bromide marked by red fluorescence, suggesting the bactericidal activity (Figure 3, panel D). The untreated control cells showed green fluorescence (Figure 3, panel C). Finally, the same fraction became bacteriostatic at 16 μg/mL against *S. epidermidis* ATCC 35984 (data not shown). 

To further investigate the antimicrobial activity of MC_A3, the synergistic interaction with oxacillin against MRSA was determined using the checkerboard technique. After 24 h of incubation, we analyzed the results to look for the best combination of MC_A3 and oxacillin. The total absence of growth was observed in the wells of the checkerboard plates with the combination between 2 µg/mL MC_A3 and 0.25 µg/mL oxacillin (MIC value for oxacillin alone was 10 μg/mL), with a decrease by half of the MIC value of MC_A3. The Fractional Inhibitory Concentration (FIC) value of 0.5 was indicative of an additive effect for MC_A3 and oxacillin. Moreover, 2D-NMR investigation of the most active last fraction MC_A3 revealed the presence of endoperoxide G3 as the main component together with ursolic acid [16] besides other aliphatic compounds present in trace. Further studies are, however necessary to assess if this activity can be ascribed to the single components or their mixture.

### 2.3. Fractionation of MC_B and NMR Characterization of Alkylphloroglucinol Glucosides

**Figure 4:**
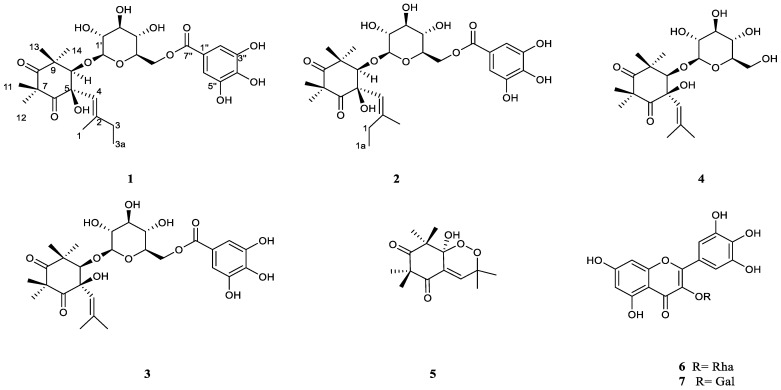
Chemical structure of compounds **1–7**, isolated from leaves of *M. communis*.

The MC_B fraction was purified by HPLC obtaining four related pure compounds (**1**–**4**, Figure 4), three of them isolated for the first time.

Compound **1** showed a molecular formula C_28_H_37_O_13_, which is in good agreement with the HRESI-MS and ^13^C-NMR data (Table 1, Figure S1, see Supplementary Materials). The ^1^H NMR spectrum of compound **1** showed typical resonances of a galloyl moiety as a singlet at δ_H_ 7.07 (δ_C_ 110.7).

An olefinic proton at δ_H_ 5.08 (δ_C_ 116.8), an oxymethine proton at δ_H_ 3.90 (δ_C_ 88.3), one allylic methyl (δ_H_ 1.75), as well as four quaternary methyls at δ_H_ 1.30, 1.22, 1.21, 1.19 were also evident in the ^1^H NMR spectrum, besides other signals in the region between 3.00 and 4.60 ppm that suggested the presence of a sugar moiety. Finally, in the aliphatic region of ^1^H NMR spectrum, the presence of a quartet at δ_H_ 1.98 and of a triplet at δ_H_ 0.93 suggested the presence of an ethyl moiety. The ^13^C NMR showed 28 carbons identified, on the basis of the HSQC experiment (Figure S2), as six methyls, two methylenes, nine methines, and eleven tetrasubstituted carbons, two of them identified as ketone carbonyls (δ_C_ 210.8 and 216.4). The sequence of the glycosidic moiety was established based on heteronuclear two-bond correlations shown in the H2BC spectrum and the data, supported by the HSQC-TOCSY experiment, suggested the presence of a glucose moiety. The coupling constant value of the anomeric proton (*J* = 8.1 Hz) allowed a β configuration for the C-1′ carbon to be assigned. The downfield shifted values of the H-6′ doublet of doublets of glucose (δ_H_ 4.51/4.47, δ_C_ 65.2) and the correlations of these protons with the carboxyl carbon at δ_C_ 168.8, allowed the localization of the galloyl group at the C-6 carbon of the sugar. The structure of metabolite **1** was confirmed by the HSQC and CIGAR-HMBC data, as reported in Table 1.

**Figure 5:**
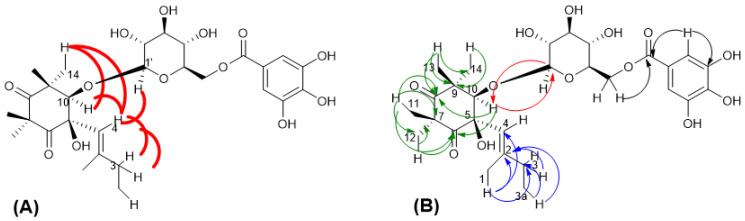
Selected NOESY correlation (**A**) and H–C long-range correlations (**B**) of compound **1** evidenced in and HMBC.

In the CIGAR-HMBC (Figure 5B), the anomeric proton at δ_H_ 4.58 showed cross peaks with the C-10 (δ_C_ 88.3), in turn correlated with H-13 and H-14 methyls. These last methyls were heterocorrelated with the C-10 and the carbonyl carbon at δ_C_ 216.4 (C-8) in turn heterocorrelated with the methyls 11 and 12. All these data (Table 1) are in agreement with the dihydroxylated cyclohexadione moiety [20] linked to a C5 unit. On the other hand, the presence of bands at 2930, 1742, 1731, 1605, cm^−1^ in the IR spectrum is in good accordance with data reported for related compounds isolated from *M. communis* [14]. The ^1^H-spin system and ^13^C multiplicities allowed us to characterize this latter unit: the *cis* configuration of the glycol moiety was assigned based on the observation of NOE among the vinylic proton H-4 and the oxymethine H-10 proton, the H-14 methyl and the methylene group (Figure 5A). Finally, NOE between vinylic proton H-4 and the anomeric proton was also evident. This structure was confirmed by the ESI Q-TOF MS/MS analysis: the tandem MS analysis of the quasimolecular ion [M-H]^−^ at *m*/*z* 581.2219 produced the fragment ion at *m*/*z* 169.0325, arising from the loss of galloyl moiety and one at *m*/*z* 124.0346, corresponding to the loss of CO_2_ molecules from the latter. Another fragment ion at *m*/*z* 271.0609 is due to the cross-ring fragmentation of the galloyl-glucose moiety as already reported by Taamalli et al. (2013) [21] for gallomyrtucommulone C. Based on the analysis of spectroscopic evidence, the chemical structure of alkylphloroglucinol **1** has been elucidated and identified as gallomyrtucommulone G, structurally related to galloylated alkylphloroglucinol glucosides reported by Appendino et al. (2006) [20].

Compound **2** was identified as *Z*-isomer at double bond of compound **1** with a molecular formula C_28_H_37_O_13_ in good agreement with the quasimolecular ion [M-H]^−^ at *m*/*z* 581.2197 revealed in the HR-ESI spectrum. The NMR spectra of **2** and **1** showed the same ^1^H spin systems and ^13^C multiplicities except for the chemical shift values of vinyl moiety (Figure S3). In fact, in compound **2,** the methyl triplet and methylene protons resonated at δ_H_ 0.97 and δ_H_ 2.25, respectively (Table 1, Figure S4).

These last signals showed heterocorrelations with both olefinic carbon at δ_C_ 118.5 (C-4) and δ_C_ 150.4 (C-2) in turn heterocorrelated with allyl methyl at δ_H_ 1.68. All others HMBC correlations (Figure S5, Table 1) allowed to identify the compound 2 as gallomyrtucommulone G. It is worth mentioning that this is the third report that describes the presence of galloylated alkylphloroglucinol glucosides in plants belonging to Myrtaceae family. Besides the gallomyrtucommulones A–D reported from *M. communis* [20], related metabolites, namely gallomyrtucommulones E–F, were described as constituents of methanolic extract of *Callistemon citrinus* leaves and stems [16].

**Figure 6:**
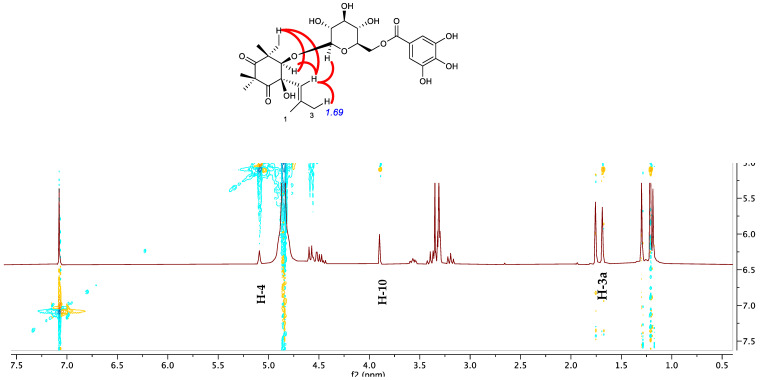
Key nOe correlation of compound **3** (top); expanded region of NOESY experiment of compound **3**.

NMR data of compound **3** are in good agreement with those described for gallomyrtucommulone D, reported by Appendino et al. (2006) [20]. However, the careful inspection of NMR data, especially NOESY experiment (Figure 6), suggested a *cis* configuration of glycol moiety as indicated by observation of nOe correlations between the vinylic proton H-4 with oxymethine proton H-10, the allylic methyl (δ_H_ 1.69) and the methyl singlet 14-CH_3_.

Compound **4** showed a molecular formula C_20_H_32_O_9_ in good agreement with the ^13^C-NMR data and the quasimolecular ions [M-H]^−^ at *m*/*z* 415.2052 and [M-H + HCOOH]^−^ at *m*/*z* 461.2079 revealed in the HR-ESI spectrum. The ^1^H-NMR spectrum (Figure S6, Table 2) of this compound, in respect to the related ones, showed the lack of singlet typical of gallic acid and the upfield shift of methylene glucose moiety at δ_H_ 3.65/3.87 (δ_C_ 62.8). The remaining signals as well as inspections of HSQC and HMBC correlations allowed the characterization of the new compound **4**, namely myrtucommulonoside (Figure 4), reported for the first time as component of *M. communis* leaves.

The NMR data of compound **5** are in good agreement with those reported in the literature for endopreroxide G3 [22], an antimalarial hormone presumably derived from the enolized trienone intermediate and so related with the gallomyrtucommulones G and H (**1**–**2**) described above. Finally, myricetin-3-*O*-rhamnoside (**6**) and myricetin-3-*O*-galactoside (**7**), already reported as components of *M. communis* leaves [21], have been also isolated from MC_B fraction. 

### 2.4. Fractionation of MC_B Antimicrobial Assessment 

**Figure 7:**
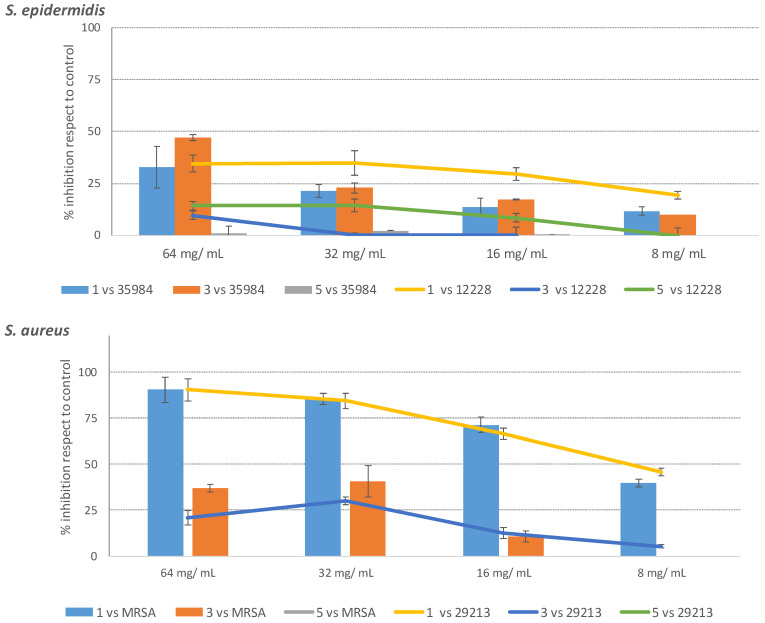
Purification and antimicrobial assessment of compounds (**1**, **3**, **5**) against *S. epidermidis* and *S aureus* strains.

The compounds **1**, **3** and **5** obtained from MC_B purification have been further investigated for their biological activities against both *S. epidermidis* and *S. aureus* (Figure 7). Compounds **2** and **4** were not tested due to insufficient material. Results highlighted that only gallomyrtucommulones G (**1**) and D (**3**) preserved their biological activity. In particular, the most active compound (**1**), provided strong activity against both *S. aureus* ATCC strains until 16 μg/mL (>65%, Figure 7). On the contrary, compound **3** showed the best activity only at 64 μg/mL (Figure 7) against MRSA (40%). Finally, both compounds turned out to be scarcely active on both strains of *S. epidermidis*, while endopreroxide G3 (**5**) was inactive.

The antimicrobial activity of MC_A3 and compound **1** turned out to be of great interest. We demonstrated that both were able to selectively reduce the growth of the pathogenic strains of *S. aureus* at a concentration that turned out to be ineffective on the skin microbiota component *S. epidermidis*. However, MC_A3 displayed a different action on the strain of *S. epidermidis* biofilm producer, since it was able to reduce bacterial growth even though at a higher concentration compared with *S. aureus*.

The results reported herein are of strong interest since the discovery of new molecules or enriched fractions selectively active on the skin microbiota components can pave the way for the realization of targeted therapy preserving the commensal bacteria, as *S. epidermidis*. Further studies could be addressed to investigate a possible involvement of these compounds in the regulation of the quorum sensing (QS) system accessory gene regulator (*agr)* within the *Staphylococcal* species [23,24].

## 3. Materials and Methods

### 3.1. Plant Material

Leaves of *M. communis* were collected at the flowering stage (April 2016) in the site of Cardedu (Sardinia, Italy, 39°49′52.3″ N 9°20′31.1″ E-693 m a.s.l.). A voucher specimen (CAG 514) has been deposited at the General Herbarium of the Department of Life and Environmental Sciences, University of Cagliari (Italy). *M. communis* is not protected by local or international regulations; therefore, no specific permission was required for its collection. The plant raw materials were dried in a ventilated stove at 40 °C to constant weight, powdered with liquid nitrogen, and stored at −20 °C until next analysis.

### 3.2. General Chromatographic Procedures

The isolation of metabolites was conducted with the aid of different chromatographic techniques. Analytical thin-layer chromatographies (TLC) were carried out on plates impregnated with silica Merck Kieselgel 60 F_254_ thickness 0.25 mm. The plates were visualized by UV light or by spraying solution H_2_SO_4_-AcOH-H_2_O (1:20:4), followed by heating in the stove for 1 min at 120 °C. The column chromatographies (CC) were performed using Merck Kieselgel 60 (40–63 µm) or Amberlite XAD-4 as stationary phase. The preparative HPLC apparatus consisted of Knauer Smartline 31/40 module equipped with Knauer Smartline 1000 pump, UV detector and PC Crom-Gate software (Knauer, Berlin, Germany). Preparative HPLC was performed using Hydro RP-18 (10 lm, 250 10.0 mm i.d., Phenomenex, Torrance, CA, USA).

### 3.3. NMR Experiments

NMR spectra were recorded at 25 °C on 300.03 MHz for ^1^H and 75.45 MHz for ^13^C on a Bruker AVANCE II 300 MHz NMR Spectrometer Fourier transform in CD_3_OD or CDCl3 (Bruker, Billerica, MA, USA). Chemical shifts are reported in δ (ppm) and referenced to the residual solvent signal; *J* (coupling constant) is given in Hz.^1^H-NMR spectra were acquired over a spectral window from 14 to −2 ppm, with 1.0 s relaxation delay, 1.70 s acquisition time (AQ), and 90° pulse width = 13.8 μs. The initial matrix was zero-filled to 64 K. ^13^C-NMR spectra were recorded in ^1^H broadband decoupling mode, over a spectral window from 235 to −15 ppm, 1.5 s relaxation delay, 90° pulse width = 9.50 μs, and AQ = 0.9 s. The number of scans for both ^1^H and ^13^C-NMR experiments were chosen, depending on the concentration of the samples. With regard to the homonuclear and heteronuclear 2D-NMR experiments, the data points, number of scans, and increments were adjusted according to the sample concentrations. Correlation spectroscopy (COSY) spectra were recorded with gradient-enhanced sequence at spectral widths of 3000 Hz in both f2 and f1 domains; the relaxation delays were of 1.0 s. The total correlation spectroscopy (TOCSY) experiments were performed in the phase-sensitive mode with a mixing time of 90 ms. The spectral width was 3000 Hz. Nuclear Overhauser effect spectroscopy (NOESY) experiments were performed in the phase-sensitive mode. The mixing time was 500 ms, and the spectral width was 3000 Hz. For all the homonuclear experiments, the initial matrix of 512 × 512 data points was zero-filled to give a final matrix of 1 k × 1 k points. Proton-detected heteronuclear correlations were also measured. Heteronuclear single-quantum coherence (HSQC) experiments (optimized for 1J (H,C) = 140 Hz) were performed in the phase-sensitive mode with field gradient. The spectral width was 12,000 Hz in f1 (^13^C) and 3000 Hz in f2 (^1^H), and had 1.0 s relaxation delay; the matrix of 1 k × 1 k data points was zero-filled to give a final matrix of 2 k × 2 k points. Heteronuclear 2 bond correlation (H2BC) spectra were obtained with T = 30.0 ms and a relaxation delay of 1.0 s; the third order low-pass filter was set for 130 < ^1^J _(C,H)_ < 165 Hz. A heteronuclear multiple bond coherence (HMBC) experiment (optimized for ^1^J _(H,C)_ = 8 Hz) was performed in the absolute value mode with field gradient; typically, ^1^H–^13^C gHMBC were acquired with spectral width of 18,000 Hz in f1 (^13^C) and 3000 Hz in f2 (^1^H) and 1.0 s of relaxation delay; the matrix of 1 k × 1 k data points was zero-filled to give a final matrix of 4 k × 4 k points. Constant time inverse-detected gradient accordion rescaled heteronuclear multiple bond correlation spectroscopy (CIGAR–HMBC) spectra (8 > ^n^J _(H,C)_ > 5) were acquired with the same spectral width used for HMBC. Heteronuclear single-quantum coherence-total correlation spectroscopy (HSQC-TOCSY) experiments were optimized for nJ (H, C) = 8 Hz, with a mixing time of 90 ms.

### 3.4. ESI MS Analyses

The exact masses were measured using a UPLC-QTOF ESI (Waters Xevo G2, Manchester, UK) high-resolution mass spectrometer (HRESI-TOFMS). Electrospray ionization in the negative polarity mode (ESI^−^) was used with the following settings: sample probe capillary voltage 3000 V, sampling cone voltage 15 V; source temperature 120 °C, and desolvation temperature 250 °C. Desolvation and cone gas with flow rates of 850 and 10 L/h were used, respectively. The raw data files were processed using MassLynx version 4.1 (Waters Inc. Milford, MA, USA, 2013). Gallomyrtucommulone G (**1**): HRMS (ESI) calcd for C_28_H_37_O_13_ [M-H]^−^: 581.2234, found: 581.2219. Gallomyrtucommulone H (**2**): HRMS (ESI) calcd for C_28_H_37_O_13_ [M-H]^−^: 581.2234, found: 581.2197. Myrtucommulonoside (**4**): HRMS (ESI) calcd for C_21_H_33_O_11_ [M-H + HCOOH]^−^: 461.2023, found: 461.2079.

### 3.5. IR Analyses

The IR spectra were determined in the CHCl_3_ using a FT/IR-4700 FT-IR Spectrometer (Jasco, Tokyo, Japan). Gallomyrtucommulone G (**1**): IR ν_max_ cm^−1^ 3066, 2991, 2930, 1789, 1742, 1731, 1605, 1592. Gallomyrtucommulone H (**2**): IR ν_max_ cm^−1^ 3068, 2989, 1789, 1744, 1603, 1550. Myrtucommulonoside (**4**): IR ν_max_ cm^−1^ 2986, 2930, 2840, 2349, 1739, 1706, 1603.

### 3.6. Hydroalcoholic Extraction of M. communis Leaves and Compounds Purification

Dried leaf material (10 g) was powder and extracted by ultrasound assisted extraction (Branson 3800 MH) for 40 min each and three cycles with H_2_O:CH_3_OH (1:1) solution (260 mL). The flask was centrifuged at 4800 rpm (Beckman, GS-15R) for 10 min at 4 °C. Subsequently, after centrifugation, the extract was filtered on Whatman paper and concentrated under vacuum, obtaining a dried crude extract (3.78 g).

The dried crude extract dissolved in H_2_O was chromatographed on Amberlite XAD-4, eluting first with water and then with methanol. The enriched organic fraction (696.1 mg) was chromatographed through Flash CC SiO_2_, eluting with the lower phase of CHCl_3_/CH_3_OH/H_2_O (13:7:4), to afford three fractions (MC_A–MC_C)

Fraction MC_A (208.2 mg) was chromatographed on Sephadex LH-20 column, using Hexane: CHCl_3_: CH_3_OH (5:1:1) as mobile phase, in turn to have three fractions MC_A1, MC_A2, MC_A3.

Fraction MC_B (92.8 mg), subjected to HPLC using H_2_O:CH_3_OH (1:1) as mobile phase, gave pure compounds **1** (5 mg), **2** (1.1 mg), **3** (7.2 mg), **4** (1.7 mg) and **5** (2.4 mg).

Fraction MC_C chromatographed on silica gel TLC (1 mm) eluting with the organic phase of biphasic solution CHCl_3_: MeOH: Me_2_CO: H_2_O 13:7:1:3) gave two spots identified as compounds **6** (40 mg) and **7** (7 mg).

### 3.7. Antimicrobial Test

#### 3.7.1. Microorganism and Growth Conditions

*S. aureus* ATCC 29213, *S. aureus* ATCC 43300, *S. epidermidis* ATCC 12228 and *S. epidermidis* ATCC 35984, were obtained from the American Type Culture Collection (Rockville, MD, USA). All the strains were cultured in tryptic soya broth (TSB, OXOID) under aerobic conditions at 37 °C for 24 h on an orbital shaker at 200 rpm. Each tested compound was dissolved in ethanol (Sigma, Milan, Italy) and diluted in TSB to give a stock solution.

#### 3.7.2. Susceptibility Assays on Bacteria Planktonic Cells 

Minimal inhibitory concentrations (MIC) of the tested compounds were determined in specific medium by the broth micro-dilution assay, according to the European Committee on Antimicrobial Susceptibility Testing (EUCAST version 7.1, 7 June 2017), as previously reported [25]. Negative control wells were set to contain bacteria in Mueller–Hinton broth plus the amount of vehicle (ethanol) used to dilute each compound. Positive controls included vancomycin (2 μg/mL) and oxacillin (2 μg/mL). The MIC was defined as the lowest concentration of drug that caused a total inhibition of microbial growth after 24 h incubation time at 37 °C. Minimum bactericidal concentration (MBC) was obtained by plating 10 μL of the medium removed from the wells in the microtiter plates where no growth was observed after 24 h of incubation at 37 °C (MIC) and was then inoculated on tryptic soya agar (TSA, OXOID), incubated for further 24 h at 37 °C. MBC was defined as the minimum concentration of antimicrobial capable of inactivating more than 99.99% of the bacteria present, causing ≥ 3log10 reduction in colony count from the starting inoculum plated. 

#### 3.7.3. Live/Dead Imaging

To study the bactericidal activity of MC_A3, live/dead staining technique was used [26]. *S. aureus* MRSA were treated with MC_A3 at MIC concentration as above reported. Cells were harvested by centrifugation after 24 h and stained with solution of acridine orange and ethidium bromide (100 μg/mL) and kept in dark for 30 min. Cells were harvested and washed twice with PBS to remove the excess stains. The cells were then observed at 20× magnification (Iris Digita System, Twin Helix, Italy).

#### 3.7.4. Checkerboard Method 

The interaction between compound MC_A3 and oxacillin against MRSA was evaluated by the checkerboard method in 96-well microtiter plates containing Mueller-Hinton broth. Briefly, MC_A3 and oxacillin were serially diluted along the y and x axes, respectively. The final concentration ranged from 0.03 to 10 μg/mL for oxacillin and from 0.5 to 8 μg/mL (0.5, 1, 2, 4, 8 μg/mL) for MC_A3. The checkerboard plates were inoculated with bacteria at an approximate concentration of 10^5^ × CFU/mL and incubated at 37 °C for 24 h, following which bacterial growth was assessed visually and the turbidity measured by microplate reader at 595 nm. The Fractional Inhibitory Concentration (FIC) index for each combination was calculated as follows: FIC index = FIC of MC_A3 + FIC of oxacillin, where FIC of MC_A3 (or oxacillin) was defined as the ratio of MIC of MC_A3 (or oxacillin) in combination and MIC of MC_A3 (or oxacillin) alone. The FIC index values were interpreted as follows: ≤0.5, synergistic; >0.5 to ≤1.0, additive; >1.0 to ≤2.0, indifferent; and >2.0, antagonistic effects [27].

## 4. Conclusions

Here we report the bio-guided investigation of glycosidic fractions of *M. communis* growing in the Sardinia island. This experimental approach led us to isolate and characterize for the first time four alkylphloroglucinol glycosides, three of them gallomyrtucommulones G–H (**1**–**2**), and myrtucommulonoside (**4**). Despite the wide distribution of oligomeric nonprenylated phologlucinols in Myrtaceae, the results reported herein represent only the third report regarding the presence of galloylated alkylphloroglucinol glucosides in plants belonging to Myrtaceae family. From a biological point of view, the results are of strong interest. We demonstrated that MC_A3 and 1 display a selective antimicrobial activity within the Staphylococcal genus, able to contrast the pathogenic species at very low concentrations but leaving undisturbed the commensal species of *S. epidermidis*. Due to their selectivity, we can hypothesize a possible use of these molecules as topical antibacterials for superficial skin infections.

## Figures and Tables

**Table 1:** NMR data of compounds **1**–**2** in CD_3_OD^1^.

	Gallomyrtucommulone G (1)			Gallomyrtucommulone H (2)		
Position	δc (Type)	δ_H_ (*J* in Hz)	HMBC (H→C)	δc (Type)	δ_H_ (*J* in Hz)	HMBC (H→C)
1	18.0 (CH_3_)	1.75 s	2, 3, 4	27.0 (CH_2_)	2.25 q (7.5)	
1a	-	-	-	13.7 (CH_3_)	0.97 t (7.5)	2, 3
2	149.9 (C)	-	-	150.4 (C)	-	-
3	34.0 (CH_2_)	1.98 q (6.0, 9.0)	2	23.9 (CH_3_)	1.68 s	1, 2
3a	11.8 (CH_3_)	0.93 t (6.0)	2, 3			
4	116.8 (CH)	5.08 s		118.5 (CH)	5.01 s	
5	83.1 (C)	-	-	83.4 (C)	-	-
6	210.8 (C)	-	-	211.1 (C)	-	-
7	56.8 (C)	-	-	56.7 (C)	-	-
8	216.4 (C)	-	-	216.9 (C)	-	-
9	49.1 (C)	-	-	49.7 (C)	-	-
10	88.3 (CH)	3.90 s	5, 6, 8, 9	89.0 (CH)	3.86 s	5, 6, 8, 9
11	25.6 (CH_3_)	1.30 s	6, 7, 8, 12	27.0 (CH_3_)	1.30 s	6, 7, 8, 12
12	24.9 (CH_3_)	1.21 s	6, 7, 8, 11	26.1 (CH_3_)	1.21 s	6, 7, 8, 11
13	28.1 (CH_3_)	1.19 s	8, 9, 10, 14	28.4 (CH_3_)	1.18 s	8, 9, 10, 14
14	29.5 (CH_3_)	1.22 s	8, 9, 10, 13	29.34 (CH_3_)	1.22 s	8, 9, 10, 13
1′	107.0 (CH)	4.58 d (8.1)	10	107.0 (CH)	4.58 d (8.1)	10
2′	75.8 (CH)	3.20 t (8.1)		75.8 (CH)	3.20 t (8.1)	
3′	78.9 (CH)	3.38 m		78.9 (CH)	3.38 m	
4′	72.3 (CH)	3.35 ov		72.3 (CH)	3.35 ov	
5′	76.2 (CH)	3.56 ov		76.2 (CH)	3.56 ov	
6′	65.2 (CH_2_)	4.51 dd (2.4, 9.3) 4.47 dd (9.3, 11.7)	7″	65.2 (CH_2_)	4.51 dd (2.4, 9.3) 4.47 dd (9.3, 11.7)	
1″	122.1 (C)	-		122.1 (C)	-	
2″	110.7 (CH)	7.07 s	1″, 3″, 5″, 7″	110.7 (CH)	7.07 s	1″, 3″, 5″, 7″
3″	146.7 (C)	-		146.7 (C)	-	
4″	140.8 (C)	-		140.8 (C)	-	
5″	146.7 (C)	-		146.7 (C)	-	
6″	110.7 (CH)	7.07 s	1″, 3″, 5″, 7″	110.7 (CH)	7.07 s	1″, 3″, 5″, 7″
7″	168.8 (C)	-		168.8 (C)	-	

d = doublet, dd = doublet of doublet; m = multiplet; ov = overlapped; q = quartet; s = singlet; t = triplet; *J* values (Hz) are reported in brackets.

**Table 2:** NMR data of compound **4** in CD_3_OD^1^.

Position	δc (Type)	δ_H_ (*J* in Hz)	HMBC (H→C)
1	19.8 (CH_3_)	1.81 d (1.2)	2, 3, 4
2	144.6 (C)	-	-
3	27.2 (CH_3_)	1.72 d (1.5)	1, 2, 4
4	119.0 (CH)	5.14 dd (1.5, 1.2)	
5	83.8 (C)	-	-
6	210.8 (C)	-	
7	56.8 (C)	-	-
8	216.2 (C)	-	-
9	49.9 (C)	-	-
10	89.1 (CH)	4.04 s	5, 6, 8, 9, 1′
11	25.1 (CH_3_)	1.26 s	6, 7, 8, 12
12	27.6 (CH_3_)	1.32 s	6, 7, 8, 11
13	26.1 (CH_3_)	1.33 s	8, 9, 10, 14
14	28.1 (CH_3_)	1.38 s	8, 9, 10, 13
1′	106.6 (CH)	4.58 d (8.1)	10
2′	75.5 (CH)	3.17 dd (8.1, 9.0)	1′
3′	78.4 (CH)	3.34 ov	
4′	78.0 (CH)	3.26 ov	
5′	78.4 (CH)	3.54 m	
6′	62.8 (CH_2_)	3.65 dd (5.6, 11.8) 3.87 dd (1.8, 11.8)	

d = doublet, dd = doublet of doublet; ov = overlapped; s = singlet; *J* values (Hz) are reported in brackets.

## Data Availability

Not applicable.

## Supplementary Materials

The following supporting information can be downloaded at: https://www.mdpi.com/article/10.3390/molecules27207109/s1, Figure S1: ^13^C-NMR spectrum of compound **1**; Figure S2: HSQC spectra and ^1^H-NMR of compound **1**. Figure S3: ^1^H-NMR spectrum of compound **1** and **2** mixture. Figure S4: HSQC and ^1^H-NMR spectra of compounds **1** and **2** mixture. Figure S5: HMBC and ^1^H-NMR spectra of compounds **1** and **2** mixture; Figure S6: ^1^H-NMR spectrum of compound **4**.

## Sample Availability

Samples of the compounds **3**, **5**–**7** are available in our laboratory.
