# Supplementary material for: Gallomyrtucommulones G and H, New Phloroglucinol Glycosides, from Bioactive Fractions of Myrtus communis against Staphylococcus Species
Source: Molecules. 2022 Oct 21;27(20):7109. doi: 10.3390/molecules27207109 (PMC9612225; doi:10.3390/molecules27207109)
Supplement: Supplementary file 1 [file molecules-27-07109-s001.zip › molecules-1970937-supplementary.pdf]

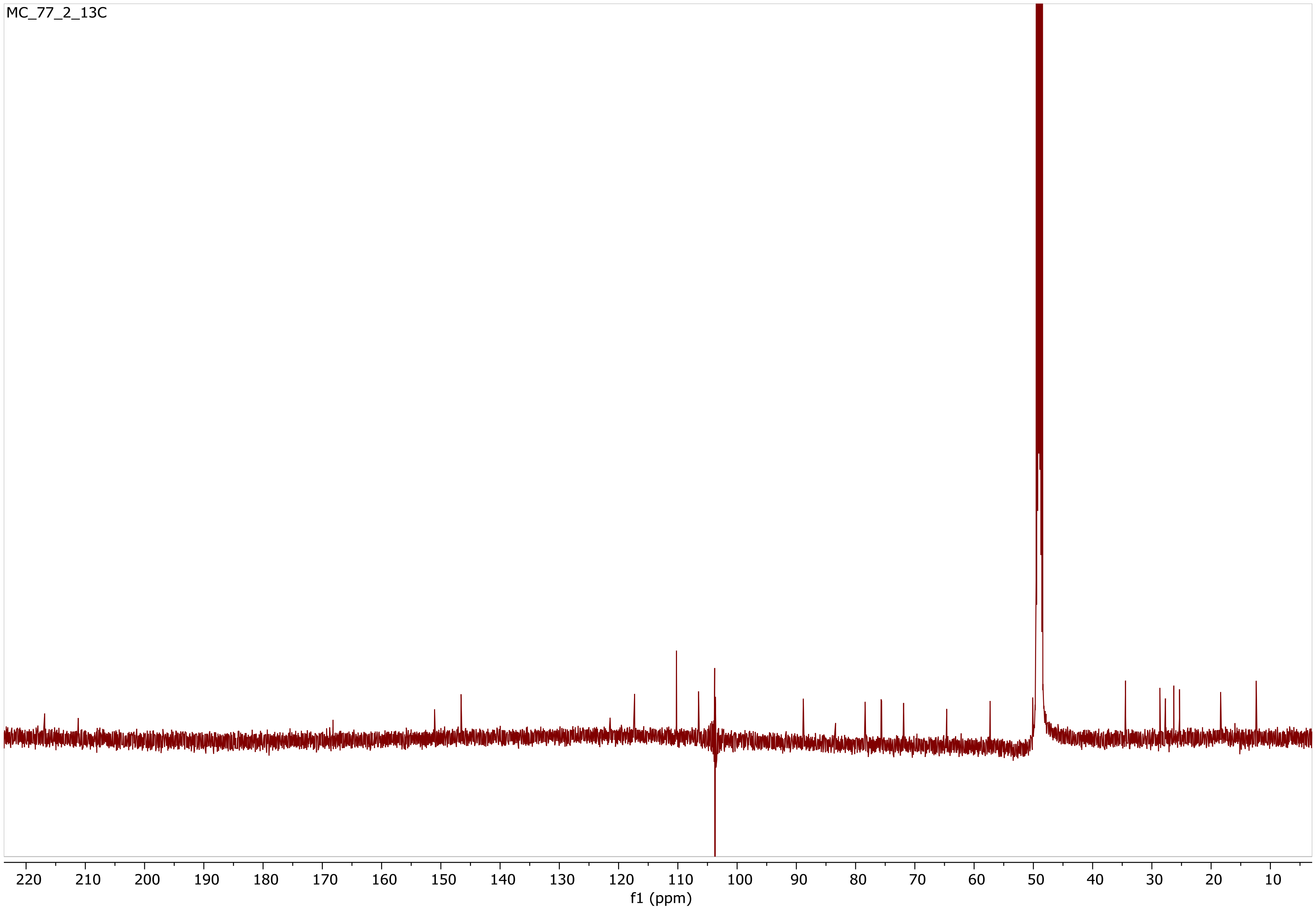

Figure S1: ^{13}C-NMR spectrum of compound **1**

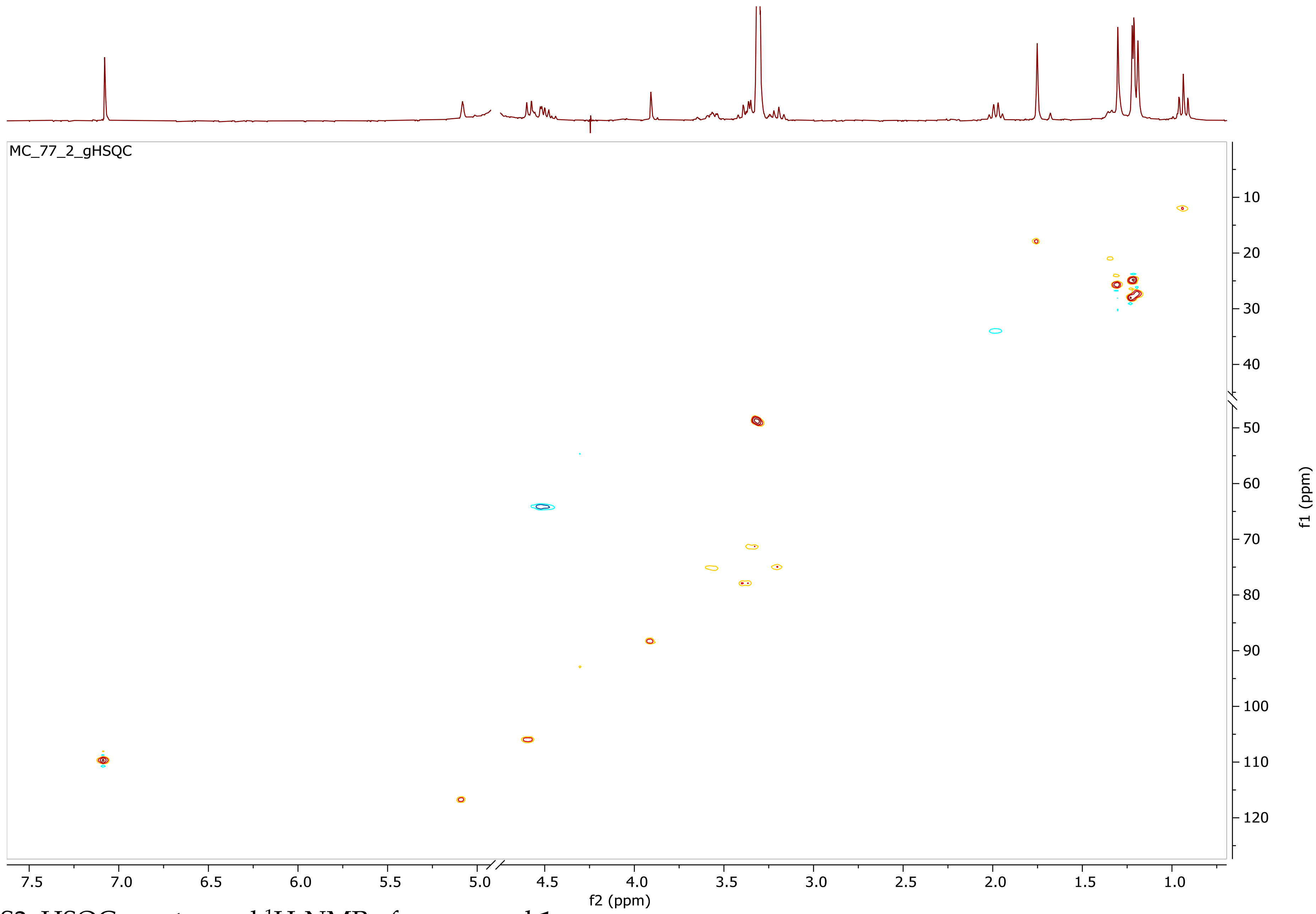

Figure S2: HSQC spectra and ^{1}H-NMR of compound **1**.

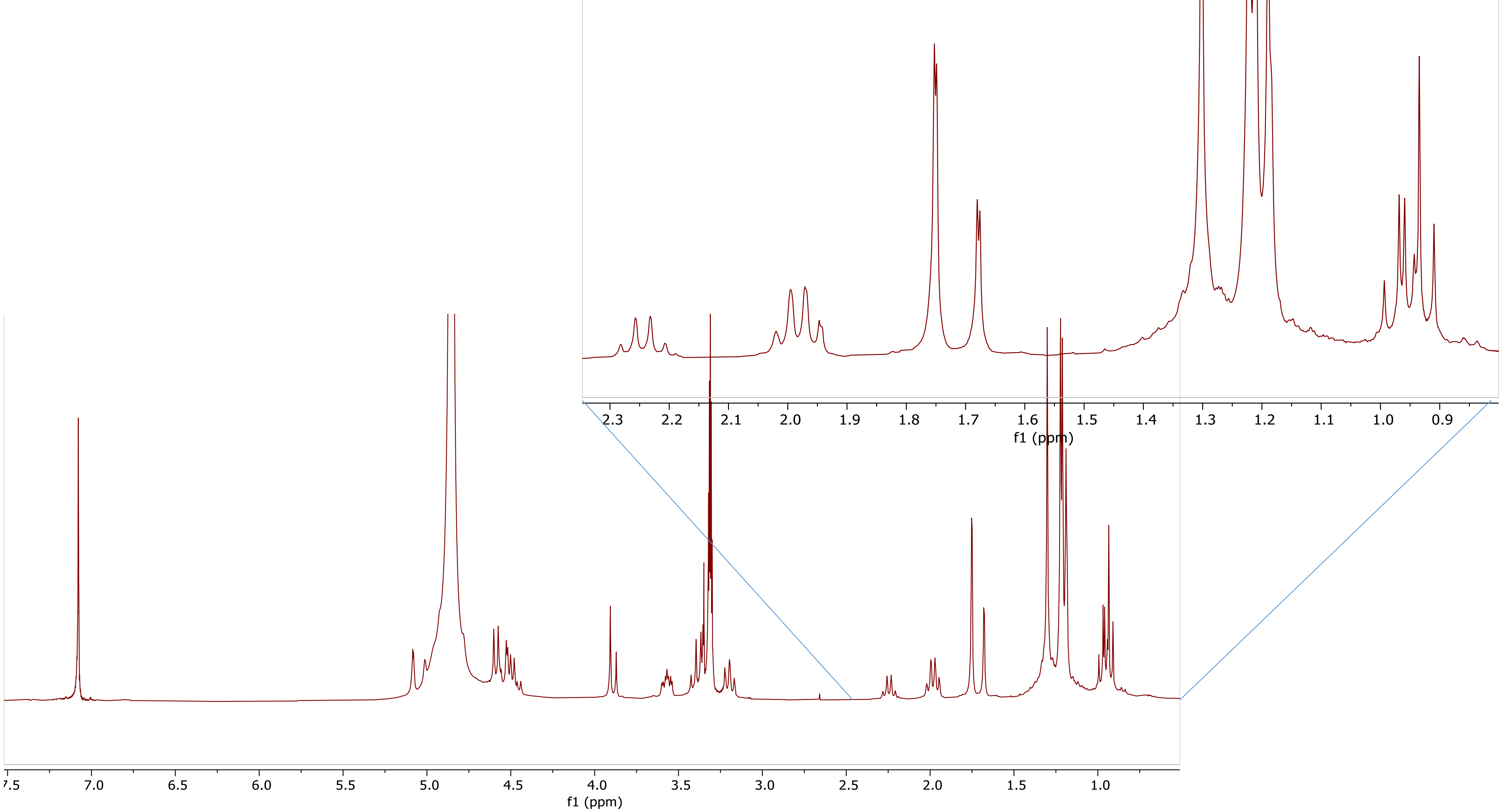

Figure S3: ^{1}H-NMR spectrum of compound **1** and **2** mixture and aliphatic expanded region

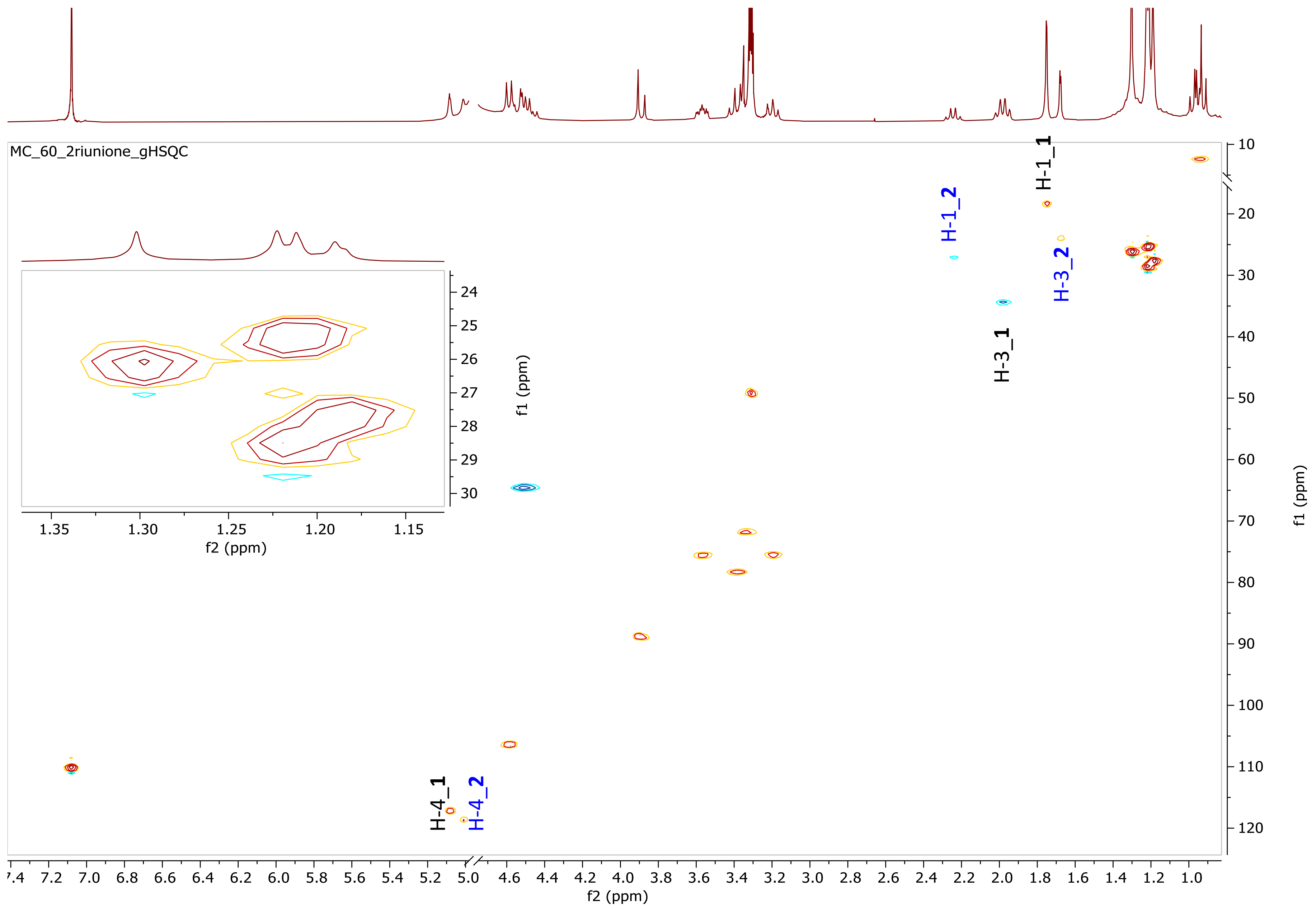

Figure S4: HSQC and ^{1}H-NMR spectra of compounds **1** and **2** mixture

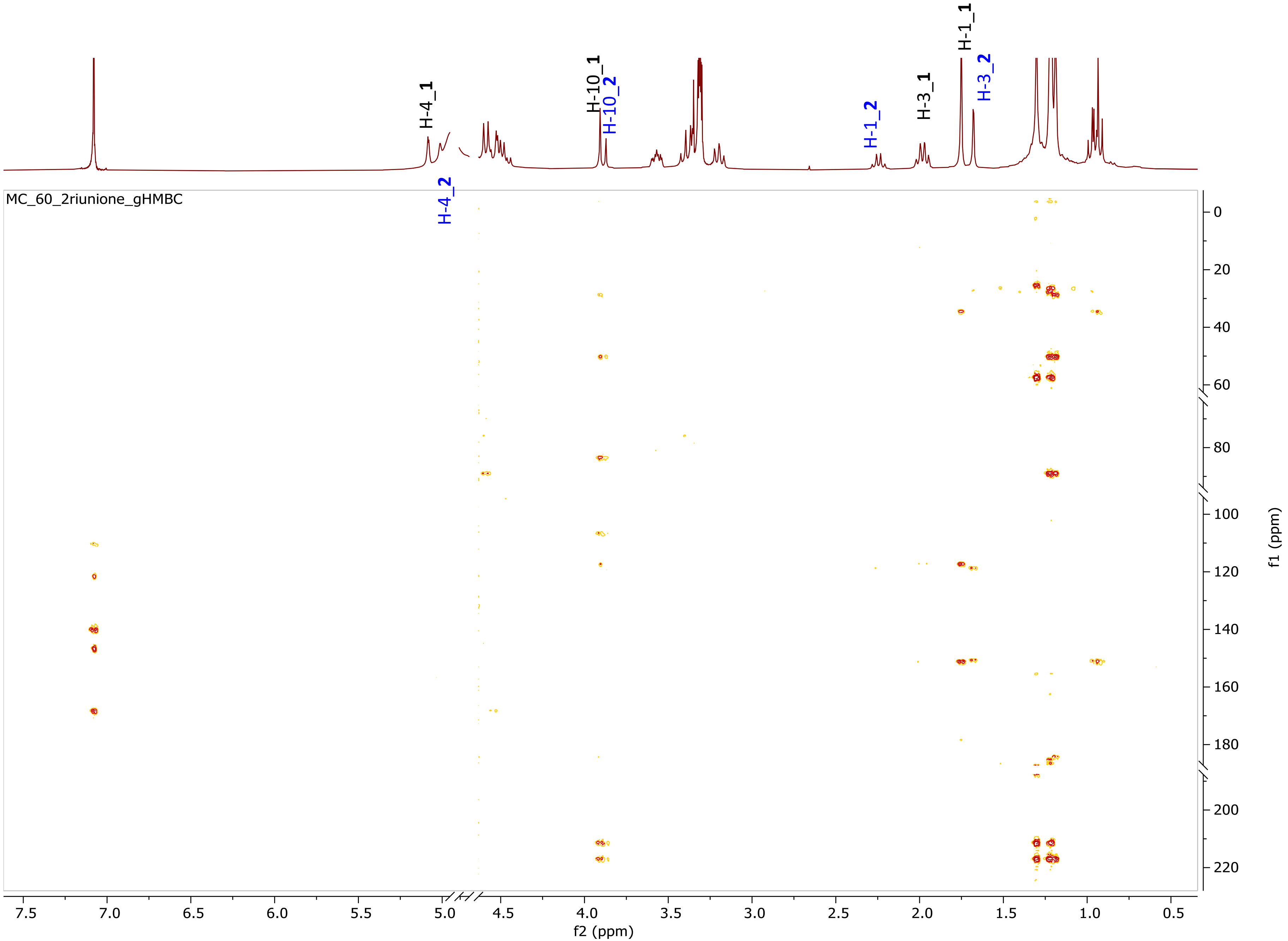

Figure S5: HMBC and ^{1}H-NMR spectra of compounds **1** and **2** mixture

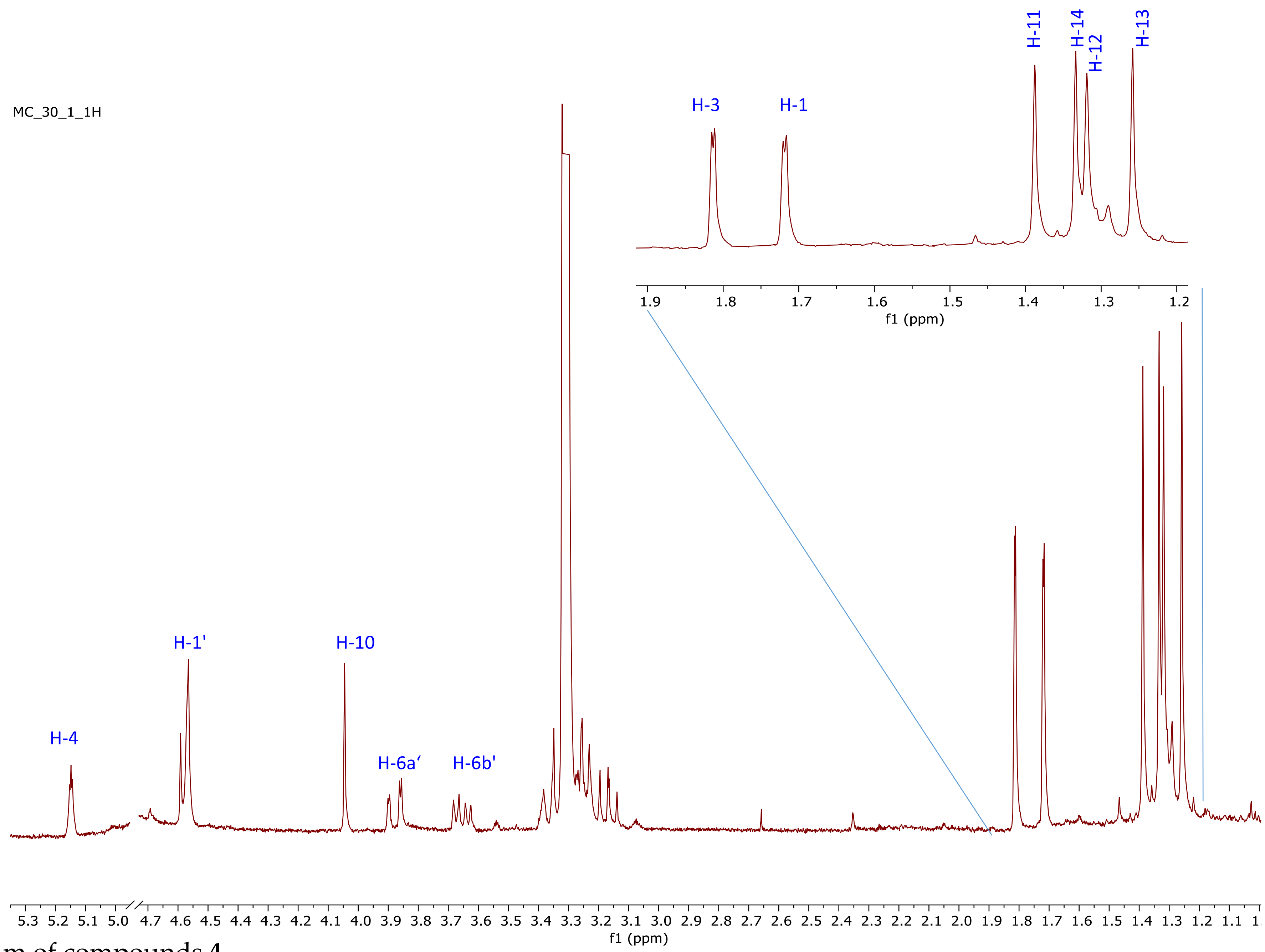

Figure S6: ^{1}H-NMR spectrum of compounds **4**
